# The Role of Poly(Methyl Methacrylate) in Management of Bone Loss and Infection in Revision Total Knee Arthroplasty: A Review

**DOI:** 10.3390/jfb11020025

**Published:** 2020-04-10

**Authors:** Leyla Hasandoost, Omar Rodriguez, Adel Alhalawani, Paul Zalzal, Emil H. Schemitsch, Stephen D. Waldman, Marcello Papini, Mark R. Towler

**Affiliations:** 1 Faculty of Engineering and Architectural Science, Biomedical Engineering Program, Ryerson University, Toronto, ON M5B 2K3, Canada; leyla.hasandoost@ryerson.ca (L.H.); swaldman@ryerson.ca (S.D.W.); mpapini@ryerson.ca (M.P.); 2 Li Ka Shing Knowledge Institute, St. Michael’s Hospital, Toronto, ON M5B 1W8, Canada; oarodrig@ryerson.ca (O.R.); adel.alhalawani@ryerson.ca (A.A.); emil.schemitsch@lhsc.on.ca (E.H.S.); 3 Department of Mechanical & Industrial Engineering, Ryerson University, Toronto, ON M5B 2K3, Canada; 4 Faculty of Health Sciences, Department of Surgery, McMaster University, Hamilton, ON L8S 4L8, Canada; paulzalzal@gmail.com; 5 Oakville Trafalgar Memorial Hospital, Oakville, ON L6J 3L7, Canada; 6 Department of Surgery, University of Western Ontario, London, ON N6A 4V2, Canada; 7 Department of Chemical Engineering, Ryerson University, Toronto, ON M5B 2K3, Canada

**Keywords:** PMMA, revision total knee arthroplasty, bone loss, infection, antibiotic-loaded bone cement

## Abstract

Poly(methyl methacrylate) (PMMA) is widely used in joint arthroplasty to secure an implant to the host bone. Complications including fracture, bone loss and infection might cause failure of total knee arthroplasty (TKA), resulting in the need for revision total knee arthroplasty (rTKA). The goals of this paper are: (1) to identify the most common complications, outside of sepsis, arising from the application of PMMA following rTKA, (2) to discuss the current applications and drawbacks of employing PMMA in managing bone loss, (3) to review the role of PMMA in addressing bone infection following complications in rTKA. Papers published between 1970 to 2018 have been considered through searching in Springer, Google Scholar, IEEE Xplore, Engineering village, PubMed and weblinks. This review considers the use of PMMA as both a bone void filler and as a spacer material in two-stage revision. To manage bone loss, PMMA is widely used to fill peripheral bone defects whose depth is less than 5 mm and covers less than 50% of the bone surface. Treatment of bone infections with PMMA is mainly for two-stage rTKA where antibiotic-loaded PMMA is inserted as a spacer. This review also shows that using antibiotic-loaded PMMA might cause complications such as toxicity to surrounding tissue, incomplete antibiotic agent release from the PMMA, roughness and bacterial colonization on the surface of PMMA. Although PMMA is the only commercial bone cement used in rTKA, there are concerns associated with using PMMA following rTKA. More research and clinical studies are needed to address these complications.

## 1. Introduction

**Figure 1:**
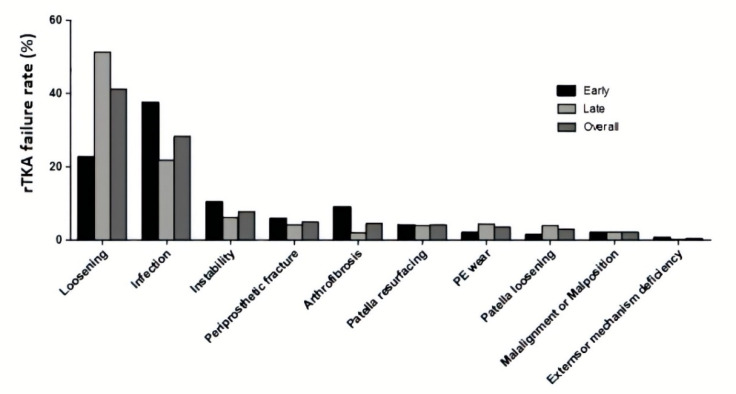
Percentage of failure of total knee arthroplasty (TKA) for different failure mechanisms into early, late and overall subgroups. Used with Permission from [4].

Failure of total knee arthroplasty (TKA) necessitates revision total knee arthroplasty (rTKA) to improve the function of the knee and to relieve patient pain [1,2]. The goal of rTKA is to address complications such as bone loss and bone infection after a failed TKA. According to a recent report by the Canadian Institute for Health Information (CIHI), 84,770 (35,945, excluding patella) rTKA surgeries were performed between 2012 and 2017 in Canada [3]; the main indications for these rTKA were infection (38.4%), instability (22.7%) and aseptic loosening (16.5%) [3]. This correlates with a United States (U.S.) study performed by Sharkey et al. [4], which investigated the causes of TKA failure by performing a systematic retrospective review on 781 rTKA surgeries between 1 July 2003 and 1 July 2012 (Figure 1). Patients were divided into two groups: early (range, 1 day to 1.97 years) and late (range, 2.01 years to 30.36 years) failure depending upon the time interval between first TKA and subsequent rTKA. Infection was found to be the main reason for rTKA in the early failure group and the average time interval between TKA failure and rTKA was 0.84 years. However, aseptic loosening was the most common reason for rTKA in the late failure group, where the average time before rTKA was 6.9 years [4].

Among the different rTKA techniques and treatments for bone loss (augmentation, metaphyseal sleeves, morselized allograft, and cementation [5,6]), PMMA cement is used when the bone defect is small (less than 5 mm depth) [7,8,9]. In an attempt to address infection, antibiotics can be added to PMMA [10,11,12,13,14,15]. In this review, papers published between 1970 and 2018 were considered, and identified through searching in Springer, Google Scholar, IEEE Xplore, Engineering village, PubMed and web links based on the topics: failure at the PMMA-bone interface, bone loss management and antibiotic-impregnated spacers in rTKA. To our knowledge, this is the first review which focuses on complications of using PMMA in addressing both bone loss and bone infection following rTKA. The aim of this review was to critique the existing literature to address the following questions:

1. What are the most common complications, outside of sepsis, regarding the use of PMMA in rTKA?
2. What are the current applications and challenges using PMMA to manage bone loss in rTKA?
3. How is PMMA used to address infection in first stage rTKA and what are the subsequent complications?

## 2. PMMA

There are many papers reviewing the chemistry, utility and clinical success of PMMA in TKA. Therefore, only a short introduction explaining the chemistry of PMMA is included.

### Chemistry of PMMA

PMMA was first applied in orthopedics in 1958 for total hip arthroplasty (THA) applications [10,16,17]. Nowadays, it is the most commonly used bone cement in both TKA and rTKA. PMMA is made of powder and liquid components; the powder usually consists of an initiator (di-benzoyl peroxide, BPO), copolymer beads, a radio-opacifier (BaSO_4_ or ZrO_2_) and sometimes antibiotics, whereas the liquid component consists of the monomer (Methyl methacrylate, MMA), a stabilizer and an activator (dimethyl-para-toluidine, DMPT) [18]. PMMA acts as a “grout” as it locks the bone and implant together mechanically, with no chemical bonding [18]. Polymerization of PMMA starts by mixing the initiator and monomer [19], an exothermic reaction which can be broken down into three steps [20,21,22]: Initiation, Propagation, and Termination:

Initiation: a chemical reaction begins by the initiator degrading, resulting in the bond cleavage or electron transfer and producing two fragments with unpaired electrons called free radicals.

Propagation: activated free radicals react with the monomer to form a new free radical. The reaction of the newly formed radicals continues until there are no more monomers or a termination reaction occurs.

Termination: there are two types of termination. One is the deletion of the monomer. The other is a combination of two active polymer chain ends or a combination of one active polymer chain end with an initiator radical or inhibitors [17,19].

## 3. Complications after rTKA

Most rTKA failures occur in the first two years following surgery [23]. Infection, aseptic loosening, and instability are categorized as the three main causes for failure following rTKA surgery [24,25,26,27]. Although infection is the principal cause of complication after rTKA, periprosthetic fracture, extensor mechanism insufficiency and stiffness increase the risk of further surgical intervention [28]. Most of the complications with PMMA, which occur in primary TKA (e.g., heat generation and volumetric shrinkage) also occur in rTKA. However, the bone stock in rTKA is often insufficient to support the hardware components using only PMMA, and other techniques such as stemmed implants, bone grafting or augments might be required to manage severe bone loss [29,30,31]. Some of the complications (outside of sepsis) which often lead to subsequent rTKA or failure of the bond between the bone-cement or component-bone interface in rTKA are discussed below.

### 3.1. Aseptic Loosening

Aseptic loosening occurs due to a failure of the bond between the bone and tibial or femoral component in the absence of infection. It can also occur when there is insufficient initial implant fixation due to weak mechanical integration between the PMMA and cancellous bone, bone resorption, damage to the bone from the heat generated during PMMA polymerization [32,33] and biological or mechanical loss of fixation over time [34]. Moreover, fragmentation of PMMA can lead to aseptic loosening at the interface. This process can also provoke a biological response and lead to osteolysis, the pathological destruction or disappearance of bone tissue [35,36].

### 3.2. Third-Body Wear

Third-body wear occurs when hard particles (such as PMMA, metal or bone) are entrapped between the femoral component and the polyethylene bearing surface [37]. Although generation of ultra-high molecular weight polyethylene (UHMWPE) particles are known to intensify osteolysis, PMMA cement particles, lead to accelerated wear and subsequent osteolysis as well [37,38,39,40,41]. Bitar et al. [38] mentioned that the biological response of wear particles were dependent on both the particle and host characteristics such as size, composition and concentration. The PMMA mantle can be degraded macroscopically due to a number of factors such as microfracture within the bulk of the PMMA mantle over time, or the movement of bone and PMMA on the articulating surfaces [39,40]. These particles may or may not be phagocytosed depending on the particle size. Niki et al. [41] reported the diameters of PMMA particles generated intraoperatively to be in the range of 250–340 µm. These particles are likely to induce a three-body wear mechanism, inducing abrasion at articulating surfaces, rather than inflammatory reactions [41]. On the other hand, small particles (≤10 µm) might inhibit osteoblastic differentiation [42].

### 3.3. Heat Generation

Exothermic polymerization of PMMA can cause bone necrosis, which results in early loosening of the implant [43,44,45]. Free radicals released after polymerization can separate the covalent C=C bonds and produce 1.4 to 1.7 × 10^8^ J/$m^{3}$ of energy in the PMMA [46]. Fukushima et al. [47] investigated heat conduction from PMMA by using a finite element method (FEM). First, the PMMA temperature was recorded during ten surgeries using a digital thermometer. The thickness of the PMMA layers was also measured from x-rays and was reported as 2.8 ± 0.6 mm. The initial temperature was measured as 32 °C and heat conduction was analyzed using the FEM. The maximum temperature was predicted as 65 °C at the PMMA and 56 °C at the bone-PMMA interface. As thermo-necrosis of bone occurs at temperatures above 53 °C [45], results indicated that the high temperature produced during polymerization might lead to bone necrosis and loosening of implanted joint prostheses. Moreover, it also indicated that the thickness of the PMMA layer affects necrosis, i.e., the thinner the PMMA, the less the necrosis [47].

### 3.4. Volumetric Shrinkage

Volumetric shrinkage is mostly due to polymerization [48]. During the process, the liquid monomer is converted to a solid polymer and the associated change in density is considered the primary cause of shrinkage [49]. The overall theoretical volume shrinkage is estimated to be up to 7% [50,51,52]. Lennon et al. [53] proposed a physical model containing a medial and lateral layer of PMMA between the bone and component surface to understand the damage accumulation around PMMA. The residual stress was approximately 4–7 MPa caused by shrinkage which could increase to more than 24 MPa due to local stress concentration caused by the presence of pores and/or if interdigitation raised the stress at the bone-PMMA interface. It was concluded that this residual stress was influenced by the temperature. Orr et al. [48] injected Palacos R^®^ (Heareaus Medical, Germany) into ring molds (60 mm × 60 mm × 3 mm). They measured the polymerization temperature and analyzed the circumferential stress of PMMA rings caused by shrinkage. The circumferential stress increased by increasing the temperature. Calculated circumferential stress of curing PMMA was in the range of 8.4–25.2 MPa for setting temperatures of 60–140 °C. The circumferential stress at 80 °C was 12.6 MPa, which was reported as the ultimate stress of PMMA [48]. Moreover, the ratio of monomer to polymer and the ambient polymerization conditions were crucial in controlling the pre-load cracking [53].

## 4. Management of Bone Defects in rTKA

For successful surgical outcomes, it is crucial to manage bone deficiencies in rTKA which require proper pre-operative evaluation and surgical planning [5]. Not only does bone deficiency restrain component alignment, but it also prevents rTKA stabilisation [7]. To categorize femoral and tibial defects in rTKA, several classifications and protocols have been introduced [7,8]. These classifications are defined so that surgeons accurately evaluate bone loss and decide which approach leads to the best treatment based on the location, size, and severity of defect in rTKA [54]. Each classification targets different aspects (appearance, size or severity) and might consider the tibia, femur or both bones [6,55]. The most frequently used classification of bone defects in rTKA is the Anderson Orthopedic Research Institute (AORI) one [6], which considers description and treatment options for bone defects in the femur and tibia separately, (Table 1) [7,56]. Below are the characteristics of the AORI classification [8,57]:

1. The terminology used for femoral and tibial defects is the same because the metaphyseal segment of both femur and tibia are similar;
2. As there is no cortical bone in the metaphyseal segment of the proximal tibia and distal femur, the common definitions which were used in most classifications (cortical /cancellous, contained/uncontained, central/peripheral) were eliminated;
3. The definitions in AORI are precise. This reduces the ambiguity when characterizing the bone defect;
4. The number of defect types in this classification is minimal to allow researchers to have sufficient cases for statistical analysis;
5. The classification provides intraoperative and postoperative radiographic data, therefore; researchers would have access to the retrospective categorization of cases.

**Figure 2:**
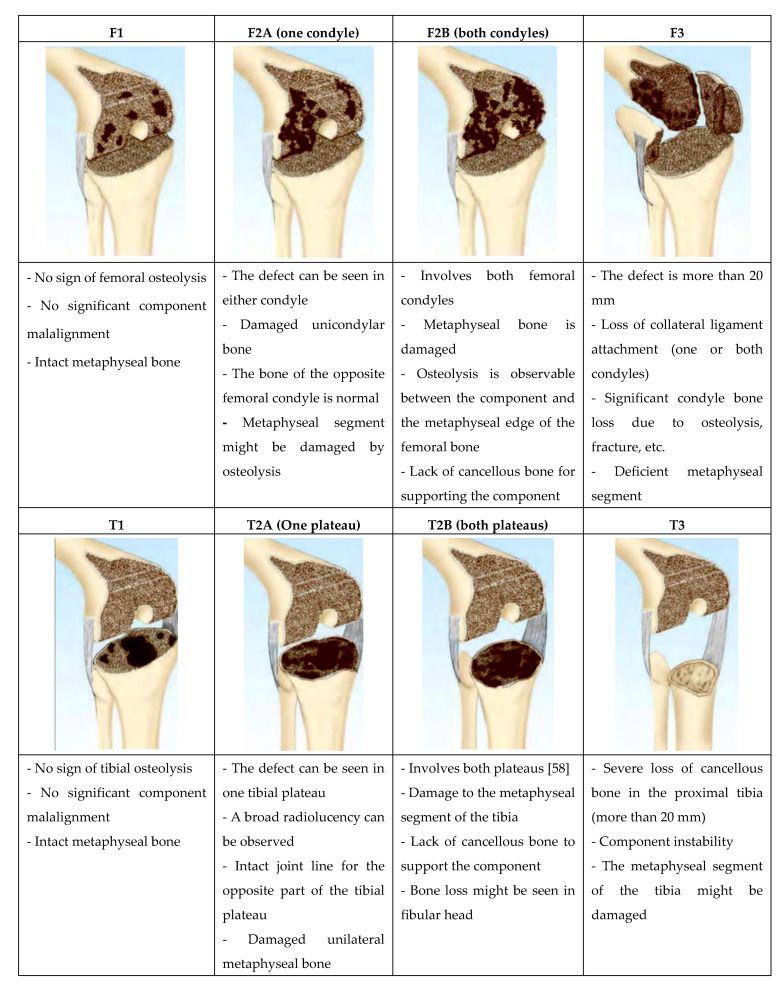
Classification and description of bone defect in the femur and tibia based on the AORI classification [8,55,58]. Used with permission from [8].

In AORI, each femoral and tibial bone defect is categorized into three types and for each, a specific treatment is suggested [6]. The following should be observed in preoperative assessment of femoral (F) or tibial (T) bone to be categorized in any of F1/T1, F2/T2, F2a/T2a, F2b/T2b and F3/T3 type of defect (Figure 2).

### 4.1. Management of F1/T1 Defect

The literature since 1984 has reported limitations in the use of PMMA for managing large bone defects [6,7,8,54,59]. Recent reports show that this limitation still exists [9,60,61,62]. PMMA is mainly used to fill peripheral bone defects having a depth of less than 5 mm and covering less than 50% of the bone surface [6,60,61,62,63,64,65]. In the case of deficiencies with 5–10 mm depth, screws can be implemented with PMMA [61]. If the component position is misaligned, screws can be used in combination with PMMA to maintain the proper stability of the component during the polymerization of PMMA [59,61]. Ritter et al. [66] examined the use of PMMA with screws for tibial defects (9 mm height) in 59 patients for a mean follow-up of 2–3 years and reported that in 27% of cases, radiolucency up to 1 mm in the bone-PMMA interface was observed. Therefore, it was concluded that screws should be used along with PMMA to fill bone defects greater than 5 mm [66].

### 4.2. Management of F2/T2 Defect

#### 4.2.1. Management of F2A

If the bone defect in one condyle is 5–10 mm deep, techniques such as morselized allografts or metal augments can be used [7,8,60]. Management of F2A defect is crucial since resection of the opposite condyle more than the proximal level, can convert F2A defect to F2B. The damaged condyle must be fixed by using a technique such as modular augmentation in order to restore the normal joint line [6].

#### 4.2.2. Management of F2B

Augmentation might be performed for both femoral condyles (distally and posteriorly) to restore the joint line [67]. Another option is using PMMA to replace the lost femoral condyle [5]. In some F2B defects, elevation of the joint line is necessary to provide motion and prevent further stiffness, so stemmed components have been recommended (with or without augmentation) [56].

##### Management of T2A

To manage T2A, a stemmed implant has been suggested as a management option in this defect. The stem might be inserted in the tibial portion to decrease the movement of the implant [68]. A small autograft, allograft or wedged tibial component would help in fixation of the implant when there is insufficient bone [69]. PMMA can also be attached to the undersurface of the tibial tray to increase fixation with an augment [70].

##### Management of T2B

Porous tantalum (Ta) tibial cones can be used in the treatment of type-2 and type-3 defects to restore the proximal tibia metaphysis [30]. When two plateaus are involved, the surgical options are a long-stemmed tibial component, a bone graft, augmentation or PMMA reinforced with screws in order to reconstruct the tibial plateau [6].

##### Stems

Stems are used to decrease strain at the bone-cement interface (distal femur or proximal tibia) by transferring the load to a larger area in order to provide additional mechanical stability for component fixation [71]. Stems can be cemented or cementless (press-fit) [72,73]. Table 2 shows a comparison of cemented and cementless stem techniques in rTKA with a minimum 24-month follow-up. 

As seen in Table 2; over time, cementless stems showed similar survivorship, and reinfection and mechanical failure rates to cemented stems. From 2008, no significant differences in failure rates were observed between cemented and cementless stems. Cementless stems are usually longer than cemented stems and press-fitted to the diaphysis of the bone, whereas cemented stems are fixed at the metaphysis of the bone [79]. There are concerns regarding the use of both cemented and cementless stems. One complication of short cemented stems is that they are unable to reach the diaphysis of the bone and therefore, in cases of severe bone loss, using short-cemented stems may result in component misalignment. Additionally, removing these stems in the future would increase the chance of bone loss [80].

### 4.3. Management of F3/T3 Defect

#### 4.3.1. Management of F3 Defect

Reconstruction in this type of defect is more complicated than the other types due to severe bone loss. Most often, this defect occurs as a result of movement of the stem along the axis of the femur. PMMA is not suggested to be used here due to the wide defect area [81]. Shrinkage of PMMA, inadequate penetration, lack of biodegradability and bioactivity, thermal necrosis and poor osteointegration restrict the use of PMMA in an F3 defect [82]. Management includes the use of structural allograft, cones or metaphyseal sleeves [8].

#### 4.3.2. Management of T3 Defect

A major portion of the proximal tibia must be replaced with a large allograft or custom-made component [67].

## 5. Management of Infection in rTKA

One of the main challenges after TKA is the risk of infection [83,84,85]. rTKA due to infection is $60,000 more expensive than revision due to the increased risk of aseptic loosening and mechanical failure [86]. In order to eradicate the infection, the patient has to stay longer in the hospital and multiple surgeries are often needed [87]. Currently, there are three options for treating infection in rTKA: (1) Debridement, antibiotics and implant retention (DAIR) (2) single-stage rTKA and (3) two-stage rTKA [88]. Discussing DAIR is out of the scope of this review paper. Therefore, two procedures (single-stage and two-stage rTKA) are discussed below.

### 5.1. Single-Stage rTKA

The first single-stage rTKAs were performed in 1976 (Hamburg, Germany) on 104 infected knees [89]. In single-stage rTKA, the infected tissue and implant are removed, along with any PMMA; remnants of the PMMA may cause severe joint pain and aseptic loosening [90,91]. Following this, rTKA is performed.

### 5.2. Two-Stage rTKA

Two-stage revision is the most commonly used method for treating infection in rTKA and involves two separate steps. In the first step, the infected implant and pre-implanted PMMA should be removed with debridement of infected bone and soft tissue. The resulting gap is then filled with an antibiotic-containing PMMA spacer [92], which releases a high concentration of antibiotics locally to treat the infection [83]. After 6–8 weeks, the PMMA spacer is removed and TKA is performed.

### 5.3. Comparison between Single-Stage and Two-Stage rTKA

There is a debate about the effectiveness of single-stage or two-stage rTKA in eradicating infection. Table 3 presents some of the literature’s conclusions on the outcomes of using either single-stage or two-stage rTKA.

From Table 3, it can be concluded that there is still controversy about whether single-stage rTKA is a better technique for patients. Lack of consistency with regards to sample size, limited number of single-stage rTKAs, period of antibiotic therapy and lack of detailed subgroup analysis are some of the limitations, which prevent a robust comparison of the effectiveness in single-stage and two-stage techniques [88,96,97]. Yan et al. mentioned two-stage rTKA as the most preferable technique in North America [97]. The Canadian Joint Replacement Registry (CJRR) reported different reasons for knee revision among 2468 procedures performed between 2016 and 2017 (Table 4).

Table 4 shows that the total failed rTKA due to infection in two-stage rTKA (stage one and two) was higher than that of the single-stage procedure. However, no details of the causes for such infections were provided in the report. Nagra et al. [88] recommended two-stage rTKA over single-stage for patients with sepsis, the presence of infection with no organism recognition, difficult pre-operative culture treatment, the appearance of sinus tract and deficient non-viable soft tissue.

### 5.4. Antibiotic-Loaded PMMA

In the case of infection, high dose antibiotic-loaded PMMA can be used in rTKA [15]. Antibiotics in the spacer should target both gram-negative and gram-positive pathogens. Thermal stability, low risk of allergy, maintaining low influence on the mechanical properties of PMMA and low binding with protein are other desired properties of antibiotic bone cement [98]. Two types of antibiotic-loaded PMMA spacers used in two-stage revision after chronically infected TKA are static (non-articulating) and dynamic (articulating) spacers [98,99].

#### 5.4.1. Static-PMMA Based Spacer

Static-PMMA spacers are made of one block of antibiotic-loaded PMMA fitted into the joint space for the purpose of releasing antibiotic drugs into the surrounding area [100,101,102]. When using a static spacer, the knee joint can only be in full extension or minimal flexion [98]. One significant drawback of the static spacer is that they lock the joint by creating a temporary arthrodesis, restricting range of motion (ROM) between the stages of revision [98,102]. It is also reported that static spacers increase bone loss [103,104] as the patient is unable to move the knee. This is explained by Wolff’s law; decreased loading on the bone results in a reduced bone density due to a decrease in the stimulus necessary for bone remodeling [105,106]. Other disadvantages include joint stiffness after rTKA, instability, wound healing problems and capsular and quadricep scarring [107]. These factors drove the development of dynamic-PMMA spacers [107].

#### 5.4.2. Static Versus Dynamic Spacers

Hsu et al. [108] compared the range of motion in static and dynamic spacers. ROM in maximum flexion before the first-stage rTKA was different for static and dynamic spacers (ROM for static spacer = 0°; ROM for dynamic spacer= 4°; P < 0.001); a two-year follow-up also showed differences in ROM for both groups (ROM for static spacer = 78°; ROM for dynamic spacer = 95°, p = 0.019) meaning that the dynamic spacer facilitated ROM more significantly than that of the static spacer. No significant difference in reinfection rate was reported for static and dynamic spacers [109,110]. However, care should be taken in using dynamic spacers in type F3 bone defect [111].

#### 5.4.3. Dynamic-PMMA Based Spacer

Dynamic spacers have shown similar eradication rates to static spacers in treating the chronic prosthetic joint infection (PJI) in rTKA while facilitating joint mobility during recovery. Several studies reported dynamic spacers improve ROM and decrease bone loss [110,112]. Two categories of intraoperatively-made dynamic spacers are cement-on-cement and metal-on-polyethylene (PROSTALAC) spacers [112].

##### Cement-on-Cement Dynamic Spacers

Cement-on-cement spacers can be either molded or pre-fabricated [98]. Molds have various sizes and shapes and can be fabricated with PMMA and a provisional component which is the same size as the original implant [112]. After filling the mold with the antibiotic-loaded PMMA, the mold is removed and the spacer substituted for the old prosthesis [112,113]. A pre-fabricated dynamic spacer is another form of cement-on-cement dynamic spacer which is made in different sizes and loaded with antibiotics [114]. The use of a pre-fabricated spacer is more economical than the molded spacers and reduces operative time [115].

##### Metal-on-Polyethylene Spacer (PROSTALAC™)

The PROSTALAC™ system consists of a metal-on-polyethylene articular surface and antibiotic-loaded PMMA (DePuy, Warsaw, IN, USA [116,117]. These can be created with commercially available mold or a homemade mold fabricated by the surgeon [118]. Gooding et al. [119] compared 115 two-stage revisions using the PROSTALAC system. The interval between two surgeries was approximately 17 weeks [119]. Following the second-stage revision, each patient was examined, first after 3 months and again at 6 months, 1 year and then yearly up to 5 to 12 years. The results showed that the use of PROSTALAC spacers led to the prevention of re-infection in 98% of cases [119].

#### 5.4.4. Concerns Regarding Use of Antibiotic-Impregnated PMMA Spacers

Several studies have reviewed both articulating and non-articulating PMMA spacers [98,111,120,121,122]. The delivery of thermosensitive antibiotics is not possible with PMMA due to the heat generated during curing of PMMA [43]. Moreover, PMMA is a non-resorbable material which must be removed in the second stage of rTKA [123]. Microorganisms tend to adhere to tissue surface and implanted biomaterials by encasing themselves in biofilm made of polysaccharide and protein [124,125]. PMMA is in direct contact with bone and blood, so plasma protein forms the conditioning film on the surface of PMMA. The initial burst release of antibiotic from PMMA occurs within 24 h, with poorly sustained elution [126,127]. If the concentration of antibiotic passes a sub-inhibitory level, the immune system might consider the PMMA as a foreign body and risk of secondary infection would increase [128]. Once the burst is over, PMMA can act as a bed for bacterial colonization (178). The incomplete drug release of PMMA might be related to the hydrophobic nature of PMMA (although carbonyl groups of PMMA create a small bond with water) [129].

Roughness is another important factor which affects the adherence of bacteria on the surface of PMMA [130]. Dantas et al. [131] concluded that an increase in PMMA roughness facilitated bacterial adhesion due to the presence of grooves and pits and protection of bacteria from shear forces. Roughness plays an important role in early plaque formation. Roughened surfaces offer a greater area for bacterial colonization [132]. Taylor et al. showed that a low roughness (Ra = 1.24 μm) increases the bacterial adhesion significantly [132]. The area covered by *P. aeruginosa* increased up to 18% when the surface of PMMA was abraded (Ra = 1.24 μm), while on a smooth surface (Ra = 0.04 μm) the area of bacterial attachment reduced to 12% [132]. Therefore, the characteristics of the surface of PMMA is crucial to avoiding bacterial resistance in the future [133].

## 6. Conclusions

PMMA is predominantly used to solve two peri-operative issues in rTKA: bone loss and bone infection. This review attempted to address three main objectives regarding employing PMMA in rTKA. The following conclusions were made:

1. Regarding the most common complications, outside of sepsis, involving the use of PMMA in rTKA. Most literature reported infection as the major cause of failure in rTKA [96,98,119]. Using PMMA also resulted in wear debris, bone necrosis and volumetric shrinkage. These might lead to complications such as tissue necrosis, instability of the implant, increased bone loss and subsequent loosening;
2. Investigating the current applications and drawbacks of using PMMA in addressing bone loss in rTKA. Literature since 1984 has mentioned limitations in using PMMA for large defects and recent reports showed no deviation on this issue [6,8,54,59,60,61,62,64]. According to the AORI classification, PMMA alone can only be used in F1/T1 defects having a depth of less than 5 mm and covering less than 50% of the bone surface [9,60,61,62,63]. We conclude that drawbacks such as crack propagation and loosening [18] restrict the use of PMMA in large bone defects.
3. Reviewing how PMMA is used to address bone infection in rTKA and what subsequent complications might result; PMMA is the standard for delivering antibiotics in infected rTKA. Antibiotic PMMA spacers are used as a treatment for patients with late chronic infection in two-stage rTKA. Although dynamic-PMMA spacers facilitate some ROM, care should be taken in using them in type-F3 bone defects. Moreover, the optimal procedure for infection eradication (single or two-stage rTKA) is still controversial in the literature [88,93,94,95,96]. Therefore, further research for guidance on single-stage vs. two-stage rTKA in managing infection is warranted. Our review demonstrated that issues such as the initial burst of antibiotic release from PMMA, with poor subsequent sustained elution, bacterial colonization on the surface of the PMMA, roughness, heat generation during polymerization and lack of porosity influence the long-term effect of PMMA-loaded antibiotics.

In conclusion, although PMMA is the only commercial bone cement used for rTKA, there are some issues of concern and therefore room for improvement. Further investigation is warranted to address complications following the use of PMMA in rTKA.

## Figures and Tables

**Table 1:** Description, characteristics and treatment options of bone deficiencies in rTKA by Anderson Orthopedic Research Institute (AORI) classification [56].

Type	Description	Characteristics	Treatment Option
1	Minor and contained cancellous bony defects	<5 mm depth	PMMA fill, morselized allograft or autograft
2A	Defects in one femoral condyle or one tibial plateau	5–10 mm depth	Morselized allograft or metal augments
		10–20 mm depth	Metal augments, metaphyseal sleeves, structural allografts
2B	Both femoral condyles or tibial plateaus are damaged	<20 mm depth	Metal augments, metaphyseal sleeves, structural allografts, custom-made prostheses, cones
3	Deficient metaphyseal segment; a bone loss that comprises a major portion of the condyle or plateau	>20 mm depth	Structural allografts, custom-made component, cones

**Table 2:** Comparison of cemented and cementless stems in rTKA.

Author	Year of Study	Number of rTKA	Remarks
Winemaker et al. [74]	1996–2003	17 cemented 15 cementless	Better early stability of cemented stems Short-term radiographic results were not affected by cementing technique
Barrack et al. [75]	Not mentioned	66 cemented 50 cementless	Higher localized pain at the end of the cementless stem (14%) in comparison with cemented stem (11%)
Fehring et al. [76]	1986–2003	107 cemented 95 press-fit	Modified Knee Society radiographic scoring system used Higher stability rate for the cemented stem (93%) in comparison with cementless (71%)
Edwards et al. [73]	1990–2010	102 cemented 126 cementless	Lower rate of radiographic failure for cementless stem. Similar reinfection rate in both stems.
Kosse et al. [77]	2008–2010	12 cemented 11 cementless	No difference in clinical outcome and micro-motion for both cemented and cementless stems
Fleischman et al. [78]	2003–2013	108 cemented 316 cementless	Similar risk of mechanical failure for both cemented and cementless stems Higher risk of failure for patients <65 years when cemented stem is used

**Table 3:** Comparison between single-stage and two-stage for treatment of infection in rTKA.

Author	Number of Studies Reviewed	Number of Single-stage and Two-stage rTKA	Outcome
Masters et al. [93]	63 studies	58 studies two-stage 4 studies single-stage 1 study mix of two rTKA	Not enough evidence to support a technique
Chew et al. [94]	12 studies (433 revision)	Not mentioned	Lack of evidence to address if single-stage is thorough enough to treat deep infection
Baker et al. [95]	122 cases	33 single-stage 162 two-stage	No statistical differences on knee function between single-stage and two-stage rTKA,
Nagra et al. [88]	796 studies	46 single-stage 185 two-stage	No significant differences in the risk of reinfection after single-stage rTKA
Kunutsor et al. [96]	118 studies	10 single-stage 108 two-stage	Single-stage revision strategy is as effective as the two-stage revision among unselected patients in general

**Table 4:** Reasons for rTKA among single-stage and two-stage rTKA (2016–2017) [3].

Reason for rTKA	Number of Failed Procedures
Infection: single-stage rTKA	288
Infection: stage one of two-stage rTKA	216
Infection: stage two of two-stage rTKA	268
Total	2468
